# UNDERSTANDING AGING AND FRAILTY WITH A PREDICTIVE NETWORK MODEL

**DOI:** 10.1093/geroni/igz038.2524

**Published:** 2019-11-08

**Authors:** Spencer Farrell, Arnold Mitnitski, Kenneth Rockwood, Andrew Rutenberg

**Affiliations:** 1 Dalhousie University, Halifax, Nova Scotia, Canada; 2 Dalhousie University, Department of Medicine, Halifax, Nova Scotia, Canada; 3 Dalhousie University, Department of Physics, Halifax, Nova Scotia, Canada

## Abstract

Health deficits are age-related binary health issues (typically self-reported disabilities) that accumulate with age. Acquiring a deficit makes an individual more frail and susceptible to other associated deficits. We model this process as a network of health deficits that interact with each other. Mortality depends on an individual’s current deficits and their age. The model is trained with self-reported data from the Canadian Study of Health and Aging (CSHA) or the National Health and Nutrition Examination Survey (NHANES). The model generates longitudinally data for synthetic-individuals with frailty trajectories and mortality resembling the observed data. We verify this by comparing the prevalence of individual deficits, correlations between deficits, and predicted death ages with test data. Our trained model performs well on all of these measures. Our model informs our understanding of aging by providing an interaction network representing the associations between pairs of deficits. Our model can generate the frailty trajectories of individuals starting from a set of deficits at a given age. This can extrapolate the trajectories of observed individuals to older ages and enables “inducing” or “treating” deficits to understand the effects of individual deficits or sets of deficits on health.

